# Impact of Preoperative Opioid Use in Lung Transplant Recipients: A Retrospective Study

**DOI:** 10.7759/cureus.90485

**Published:** 2025-08-19

**Authors:** Austin Chang, Taisuke Kaiho, Benjamin L Thomae, Yudai Miyashita, Amanda Kamar, Anne O'Boye, Arunachalam Ambalavanan, Chitaru Kurihara

**Affiliations:** 1 Thoracic Surgery, Northwestern University Feinberg School of Medicine, Chicago, USA; 2 Pulmonary and Critical Care Medicine, Northwestern University Feinberg School of Medicine, Chicago, USA; 3 Surgery, Northwestern University Feinberg School of Medicine, Chicago, USA

**Keywords:** lung transplantation, morphine equivalent (me), opioid, primary graft dysfunction (pgd), ventilator

## Abstract

Purpose

The impact of preoperative opioids on lung transplant outcomes has not been well established. This study explores survival rates and mortality risk factors in lung transplant recipients with preoperative opioid prescriptions.

Methods

A single-institutional lung transplant database (2018-2024) was used to collect data on patient characteristics, pretransplant laboratory values, and postoperative outcomes. Opioid prescriptions were normalized to morphine equivalents (MEs). Chi-squared, one-way ANOVA, Kruskal-Wallis, Kaplan-Meier, and Wilcoxon signed-rank tests were used for analysis.

Results

Among 399 lung transplant recipients, 188 (47.1%) reported opioid use within 30 days before transplantation: 115 (61.2%) had ME ≥ 0.1 mg (high-ME), and 73 (38.8%) had ME < 0.1 mg (low-ME). There was no significant difference in one-year survival between the high-ME cohort and their counterparts (83.8% vs. 86.7% (low-ME) vs. 90.5% (no ME), p = 0.86). Intensive care unit (ICU) stay (median: 10 (5-20, interquartile range (IQR)) vs. 7 (5-13, IQR, low-ME) vs. 7 (4-14, IQR, no ME) days, p = 0.046) and hospital stay (median: 21 (13-38, IQR) vs. 17 (11-31, IQR, low-ME) vs. 16 (11-27, IQR, no ME) days, p = 0.007) were significantly longer in the high-ME cohort. The high-ME cohort had a higher incidence of primary graft dysfunction (PGD) (hazard ratio, or HR 1.84 (1.17-2.89, 95% CI), p = 0.008) and PGD Grade 3 (HR 2.16 (1.18-3.97, 95% CI), p = 0.01).

Conclusions

Preoperative opioid use in lung transplant patients is an independent risk factor for post-transplant PGD risk and a predictor of prolonged hospital stay. Weaning of opioids prior to lung transplantation may limit prolonged hospital stay and ventilation use.

## Introduction

Lung transplantation is a critical treatment option for patients with end-stage lung disease, providing substantial benefits for survival and quality of life [1,2]. Although there have been advancements in lung transplantation, managing patients remains challenging, particularly regarding the role of preoperative opioid use and its effect on patient outcomes. Opioids are frequently used to reduce preoperative pain and dyspnea, and they may also help reduce anxiety and postoperative pain [3,4]. This, in turn, can mitigate cardiovascular stress responses, such as hemodynamic instability during and after surgery. However, preoperative opioid use remains controversial because of its association with opioid misuse, mortality, and an increased risk of adverse outcomes [5].

Preoperative opioid prescriptions have been linked to several postoperative complications. These patients are more likely to have concurrent benzodiazepine use, prolonged opioid consumption after surgery, and increased mortality risk [6,7]. Additionally, studies suggest that opioids may induce hyperalgesia, especially in patients with opioid tolerance, inadvertently exacerbating pain instead of alleviating it [8]. This effect may also increase the risk of postoperative delirium [9]. These opioid-related complications contribute to longer hospital stays, higher rates of 30-day readmission, and greater healthcare expenditures [10]. In solid organ transplantation, opioid use before surgery is associated with a higher risk of graft failure and mortality [11,12]. These findings highlight opioid-related risks in transplantation, but their effect on lung transplant outcomes is still not well understood. Considering that opioids may compromise respiratory drive and are often used in patients with end-stage lung disease to manage chronic pain and dyspnea [3,13], it is essential to carefully assess preoperative opioid use. A thorough evaluation is needed to develop guidelines for the proper use and tapering of opioids in lung transplant recipients.

The effects of opioid use on transplant outcomes remain unclear, especially among lung transplant recipients who already have impaired pulmonary function. We sought to evaluate survival outcomes and mortality risk factors among lung transplant recipients with preoperative opioid prescriptions, and to examine how opioid use influences postoperative recovery. Our primary objective was to identify an opioid dosage threshold associated with a higher risk of adverse post-transplant outcomes. We hypothesized that lung transplant recipients receiving high-dose opioids preoperatively face a greater mortality risk and delayed recovery. Understanding these interactions could help develop protocols that optimize patient outcomes while reducing the potential risks of opioid use.

This article was previously presented as a meeting abstract at the 2025 ISHLT Annual Meeting, on April 29, 2025.

## Materials and methods

Study design

Ethical approval for this study was obtained from the Institutional Review Board of Northwestern University (STU00212120). The Institutional Review Board waived the requirement for patient consent for data collection, given the retrospective design of this study. 

Retrospective patient data were collected from electronic medical records and stored in a lung transplant database at the Northwestern University Medical Center in Chicago, USA. Adult patients who underwent lung transplantation at our institution from January 2018 to May 2024 were included. We used primary graft dysfunction (PGD) Grade 3 to assess post-transplant outcomes because of its association with heightened short- and long-term mortality risk [14]. To evaluate the predictive value of preoperative opioid dosage for developing PGD Grade 3, a receiver operating characteristic (ROC) curve analysis was performed. This study excluded multiorgan transplant and re-transplant recipients. Data were collected on patient demographics, comorbidities, donor characteristics, preoperative laboratory values, intraoperative and postoperative outcomes, and panel reactive antibody (PRA) results. Early post-transplant complications, length of hospitalization, and survival outcomes were also analyzed.

At our institution, patients listed for lung transplantation undergo a comprehensive multidisciplinary evaluation, which includes assessment by transplant physicians, social workers, and psychologists. When clinically feasible, opioid tapering or discontinuation is attempted prior to transplantation, and individualized pain management strategies are employed to minimize perioperative opioid exposure.

Statistical analysis

Opioid prescriptions were normalized to morphine equivalent (ME) and included both outpatient prescriptions and inpatient use within 30 days prior to lung transplantation. Recipient and donor characteristics, preoperative laboratory values, and intra- and postoperative outcomes were compared between three groups of lung transplant recipients: high-ME, low-ME, and no ME. Continuous variables were analyzed using one-way ANOVA, and F-statistics were reported. Categorical variables were reported as numbers and percentages, and were compared using the Kruskal-Wallis test, with H-statistics provided. Survival outcomes were estimated using the Kaplan-Meier method, with group survival differences compared using the log-rank test. Univariate and multivariate Cox proportional hazards analyses were employed to calculate hazard ratios (HRs) for mortality. Odds ratios (ORs) for PGD were obtained using univariate and multivariate logistic regression analyses. Patients lost to follow-up were censored in the Kaplan-Meier curve. Variables with a p-value below 0.05 in Cox proportional hazards or logistic regression analyses were included in the multivariate analysis. Statistical significance was defined as p < 0.05. All statistical analyses were conducted using EZR software (Saitama Medical Center, Jichi Medical University, Saitama, Japan), a graphical user interface for R (The R Foundation for Statistical Computing, Vienna, Austria).

Definition of complication

Primary Graft Dysfunction (PGD)

PGD was defined based on the ISHLT guideline [15] and graded by PaO₂/FiO₂ ratio as follows: Grade 1, PaO₂/FiO₂ ratio >300; Grade 2, PaO₂/FiO₂ ratio 200-300; Grade 3, PaO₂/FiO₂ ratio <200. The use of extracorporeal membrane oxygenation (ECMO) for bilateral pulmonary edema on chest X-ray images was classified as Grade 3. PGD 3 was defined as any incidence of Grade 3 within the first 72 hours post-transplantation.

Acute Kidney Injury (AKI)

AKI was defined using the risk, failure, loss of kidney function, and end-stage kidney disease classification [16].

Indication for Venovenous-ECMO (VV-ECMO) After Lung Transplant

After lung transplantation, all intubated patients were treated by a multidisciplinary team, in accordance with the guidelines of the National Heart, Lung, and Blood Institute’s ARDS Network [17]. Indications for ECMO evaluation included refractory hypoxemia with PaO₂ less than 55 mmHg, pulse oximetry oxygen saturation less than 88%, and pH level less than 7.2. Patients were evaluated with lung-protective mechanical ventilation with a plateau pressure of less than 35 mmHg, neuromuscular blockade, and prone positioning, according to recommendations from the Extracorporeal Life Support Organization [18]. Central venous catheters (CVCs) were replaced every seven days, even if there were no signs of infection. New CVC replacements were performed not via wire-based exchange in the same vein, but in a different area or a new vein. Weekly surveillance cultures for all ECMO patients were used to monitor bloodstream infections simultaneously, consistent with our previous study [19].

## Results

Patient demographics

Among 399 patients who underwent lung transplantation from January 2018 to May 2024, 188 recipients (47.1%) filled opioid prescriptions within 30 days before lung transplantation (Table 1). An ROC curve and a Youden index were used to evaluate the predictive value of preoperative opioid dosage for developing PGD Grade 3. Based on this analysis, a threshold of 0.1 mg ME was determined to best predict post-transplant outcomes (AUC = 0.57 (0.49-0.66, 95% CI), specificity = 0.73, sensitivity = 0.44, Youden index = 0.17). Patients were subsequently categorized into three groups based on opioid use: high-ME (ME ≥ 0.1 mg), low-ME (ME < 0.1 mg), and no opioid use (no ME). Among the 188 lung transplant recipients who received preoperative opioid prescriptions, 115 (61.2%) had ME ≥ 0.1 mg (high-ME group), and 73 (38.8%) had ME < 0.1 mg (low-ME group). Patients in the low-ME group were significantly older (64.9 ± 7.2 years) compared to those in the high-ME group (55.5 ± 13.4 years) and the no ME group (59.2 ± 12.3 years) (p < 0.001). Gender distribution was similar across all groups. The high-ME group had significantly greater rates of pre-transplant ECMO use (32.2% vs. 1.4% (low-ME) vs. 0.9% (no ME), p < 0.001) and bilateral lung transplantation (80.9% vs. 41.1% (low-ME) vs. 59.2% (no ME), p < 0.001), higher mean lung allocation scores (74.1 ± 18.3 vs. 50.0 ± 16.3 (low-ME) vs. 47.8 ± 13.2 (no ME), p < 0.001), and higher mean composite allocation scores (31.1 ± 9.2 vs. 29.3 ± 7.2 (low-ME) vs. 24.4 ± 5.3 (no ME), p < 0.001). Regarding comorbidities, there was a lower prevalence of smoking history (39.1% vs. 56.2% (low-ME) vs. 53.1% (no ME), p = 0.03), hypertension (41.7% vs. 64.4% (low-ME) vs. 60.2% (no ME), p = 0.002), and diabetes (20.9% vs. 35.6% (low-ME) vs. 34.1% (no ME), p = 0.02) in the high-ME group.

**Table 1:** Patient characteristics of lung transplant recipients by usage of opioids Continuous data are shown as means ± standard deviation (SD) for age and laboratory data, and as medians and interquartile ranges (Q1-Q3) for days and doses. One-way analysis of variance (ANOVA) or the Kruskal-Wallis test was used to compare independent continuous variables between the groups. The chi-squared test was used to compare categorical variables. Etiology of lung failure (other): sarcoidosis, hypersensitivity pneumonitis, cystic fibrosis, bronchiectasis, obliterative bronchiolitis, bronchoalveolar carcinoma, primary ciliary dyskinesia ALT, alanine aminotransferase; AST, aspartate aminotransferase; BUN, blood urea nitrogen; COPD, chronic obstructive pulmonary disease; COVID-19, coronavirus disease 2019; ECMO; extracorporeal membrane oxygenation; HFNC, high-flow nasal cannula; INR, international normalized ratio; ME, morphine equivalent; PAH, pulmonary artery hypertension; PRA, panel reactive antibodies; WBC, white blood cell

Variable	High-ME group (n = 115)	Low-ME group (n = 73)	No ME group (n = 211)	Test statistic, F or H value	p-value
Recipient factors					
Age, years	55.5 ± 13.4	64.9 ± 7.2	59.2 ± 12.3	14	<0.001
Female	59 (51.3%)	26 (35.6%)	85 (40.3%)	5.5	0.07
Body mass index (kg/m^2^)	25.2 ± 4.4	26.2 ± 4.4	26.4 ± 4.7	2.9	0.06
Body surface area (m^2^)	1.8 ± 0.2	1.9 ± 0.2	1.9 ± 0.2	4.7	0.009
Smoking history	45 (39.1%)	41 (56.2%)	112 (53.1%)	7.3	0.03
Hypertension	48 (41.7%)	47 (64.4%)	127 (60.2%)	13	0.002
Diabetes	24 (20.9%)	26 (35.6%)	72 (34.1%)	7.2	0.02
Concurrent benzodiazepine use	65 (56.5%)	64 (87.7%)	12 (5.7%)	190	<0.001
Pre-transplant ventilator use	22 (19.1%)	5 (6.8%)	15 (7.1%)	13	0.002
Pre-transplant HFNC use	29 (25.2%)	14 (19.2%)	19 (9.0%)	16	<0.001
Pre-transplant ECMO use	37 (32.2%)	1 (1.4%)	2 (0.9%)	88	<0.001
Bilateral lung transplant	93 (80.9%)	30 (41.1%)	125 (59.2%)	32	<0.001
Lung allocation score	74.1 ± 18.3	50.0 ± 16.3	47.8 ± 13.2	72	<0.001
Composite allocation score	31.1 ± 9.2	29.3 ± 7.2	24.4 ± 5.3	14	<0.001
On the waiting list	11 (5-24)	7 (3-16)	19 (7-48)	27	<0.001
Max dose of opioids, mg					
Initial admission	10 (5-50)	10 (5-50)	10 (5-50)	0.47	0.79
30 days post-transplant	10 (5-50)	10 (5-50)	10 (5-50)	0.17	0.92
90 days post-transplant	5 (0.05-50)	0.05 (0.025-10)	0.05 (0.025-5)	6.5	0.04
1 year post-transplant	2.5 (0.05-10)	0.4 (0.05-8)	0.1 (0.05-6.75)	2.9	0.23
Etiology of lung failure					
Interstitial lung disease	32 (27.8%)	36 (49.3%)	79 (37.4%)	8.9	0.01
COVID-19	35 (30.4%)	5 (6.8%)	8 (3.8%)	52	<0.001
COPD	10 (8.7%)	17 (23.3%)	49 (23.2%)	11	0.002
PAH	4 (3.5%)	3 (4.1%)	13 (6.2%)	1.3	0.59
Other	34 (29.6%)	12 (16.4%)	62 (29.4%)	5.1	0.07
Laboratory values					
Hemoglobin (g/dL)	10.0 ± 2.1	12.1 ± 2.2	12.3 ± 2.2	44	<0.001
WBC (1,000/mm^3^)	10.4 ± 4.1	8.9 ± 2.9	9.5 ± 3.7	3.9	0.02
Platelets (1,000/mm^3^)	248.4 ± 116.7	236.2 ± 66.1	257.0 ± 86.9	1.4	0.25
Sodium (mEq/L)	139.9 ± 4.1	139.5 ± 2.8	139.4 ± 3.1	0.98	0.38
BUN (mg/dL)	18.3 ± 10.0	16.4 ± 6.7	16.6 ± 6.2	2.3	0.1
Creatinine (mg/dL)	0.70 ± 0.25	0.84 ± 0.26	0.83 ± 0.24	12	<0.001
AST (U/L)	29.1 ± 22.3	22.6 ± 9.4	25.8 ± 17.3	3.1	0.046
ALT (U/L)	22.8 ± 17.3	18.4 ± 13.0	20.4 ± 18.5	1.6	0.21
Albumin (g/dL)	3.6 ± 0.5	4.0 ± 0.6	4.0 ± 0.5	24	<0.001
Total bilirubin (mg/dL)	0.7 ± 0.8	0.6 ± 0.5	0.6 ± 0.4	3.1	0.047
INR	1.13 ± 0.25	1.06 ± 0.14	1.07 ± 0.16	5.3	0.005
PRA	60 (52.2%)	24 (32.9%)	71 (33.6%)	12	0.002
Arterial blood gas					
pH	7.39 ± 0.07	7.38 ± 0.07	7.37 ± 0.07	7	0.001
PaCO_2_ (mmHg)	49.3 ± 10.7	50.7 ± 13.4	49.2 ± 11.0	0.49	0.61
PaO_2_ (mmHg)	242.3 ± 109.2	271.9 ± 108.1	287.7 ± 99.6	7.1	<0.001
Donor					
Age (years)	33.7 ± 12.9	36.5 ± 13.4	34.6 ± 12.3	1.1	0.32
Female	43 (37.4%)	22 (30.1%)	62 (29.4%)	2.3	0.32
Cause of death					
Anoxia	47 (40.9%)	26 (35.6%)	92 (43.6%)	1.4	0.5
Head trauma	39 (33.9%)	28 (38.4%)	70 (33.2%)	0.66	0.72
Other	29 (25.2%)	19 (26.0%)	49 (23.2%)	0.3	0.85

Compared to the no ME cohort, both ME groups showed a greater frequency of concurrent benzodiazepine use (56.5% (high-ME) vs. 87.7% (low-ME) vs. 5.7% (no ME), p < 0.001) and pre-transplant high-flow nasal cannula (HFNC) use (25.2% (high-ME) vs. 19.2% (low-ME) vs. 9.0% (no ME), p < 0.001). The high-ME group had more pre-transplant ventilator use than the remaining two groups (19.1% vs. 6.8% (low-ME) vs. 7.1% (no ME), p = 0.003). Although patients in the high-ME group received greater maximum opioid doses at 90 days post-transplant (median: 5 (0.05-50, interquartile range (IQR)) vs. 0.05 (0.025-10, IQR, low-ME) vs. 0.05 (0.025-5, IQR, no ME) mg, p = 0.04), there were no significant differences in maximum opioid dose during initial admission, at 30 days post-transplant, and at one year post-transplant.

High-ME patients had significantly higher preoperative white blood cell counts (10.4 ± 4.1 vs. 8.9 ± 2.9 (low-ME) vs. 9.5 ± 3.7 (no ME) × 1000/mm³, p = 0.02) and PRA positivity rates (52.2% vs. 32.9% (low-ME) vs. 33.6% (no ME), p = 0.003), and lower hemoglobin levels (10.0 ± 2.1 vs. 12.1 ± 2.2 (low-ME) vs. 12.3 ± 2.2 (no ME) g/dL, p < 0.001), creatinine levels (0.70 ± 0.25 vs. 0.84 ± 0.26 (low-ME) vs. 0.83 ± 0.24 (no ME) mg/dL, p < 0.001), and albumin levels (3.6 ± 0.5 vs. 4.0 ± 0.6 (low-ME) vs. 4.0 ± 0.5 (no ME) g/dL, p < 0.001). Furthermore, patients in the high-ME group showed poorer preoperative arterial blood gas profiles, with lower PaO₂ levels (242.3 ± 109.2 vs. 271.9 ± 108.1 (low-ME) vs. 287.7 ± 99.6 (no ME) mmHg, p < 0.001).

Intraoperative and postoperative outcomes

Patients in the high-ME cohort experienced significantly longer median operative time (median: 6.8 (5.5-8.3, IQR) vs. 5.1 (3.4-6.6, IQR, low-ME) vs. 5.5 (4.4-7.2, IQR, no ME) hours, p < 0.001) and required more intraoperative packed red blood cells (pRBCs) transfusions (3 (1-8, IQR) vs. 0 (0-1, IQR, low-ME) vs. 0 (0-2, IQR, no ME) units, p < 0.001) (Table 2). Furthermore, the median ischemic time was significantly longer in the high-ME cohort (5.6 (4.7-6.3, IQR) vs. 4.9 (4.0-5.9, IQR, low-ME) vs. 5.0 (3.9-6.0, IQR, no ME) hours, p = 0.006). Intraoperative use of venoarterial extracorporeal membrane oxygenation (VA-ECMO) was also more prevalent among high-ME patients (80.0% vs. 52.1% (low-ME) vs. 56.4% (no ME), p < 0.001), with a significantly longer duration of VA-ECMO support during surgery (2.8 (1.5-3.5, IQR) vs. 1.3 (0-2.8, IQR, low-ME) vs. 1.7 (0-2.9, IQR, no ME) hours, p < 0.001).

**Table 2:** Intra- and post-operative outcomes of lung transplant recipients by usage of opioids Continuous data are shown as medians and interquartile ranges (Q1-Q3). One-way analysis of variance (ANOVA) was used to compare independent continuous variables between the groups. The chi-squared test was used to compare categorical variables. CLAD, chronic lung allograft dysfunction; ECMO, extracorporeal membrane oxygenation; FFP, fresh frozen plasma; ICU, intensive care unit; PGD, primary graft dysfunction; Plt, platelets; pRBCs, packed red blood cells; VA-ECMO, venoarterial-extracorporeal membrane oxygenation

Variable	High-ME group (n = 115)	Low-ME group (n = 73)	No ME group (n = 211)	Test statistic, H-value	p-value
Intra-operative outcomes					
Operative time (hours)	6.8 (5.5-8.3)	5.1 (3.4-6.6)	5.5 (4.4-7.2)	32	<0.001
Intra-op blood transfusion					
pRBCs (units)	3 (1-8)	0 (0-1)	0 (0-2)	81	<0.001
FFP (units)	0 (0-3)	0 (0-0)	0 (0-0)	53	<0.001
Plt (units)	0 (0-2)	0 (0-0)	0 (0-0)	61	<0.001
Ischemic time (hours)	5.6 (4.7-6.3)	4.9 (4.0-5.9)	5.0 (3.9-6.0)	10	0.006
VA-ECMO use	92 (80.0%)	38 (52.1%)	119 (56.4%)	22	<0.001
VA-ECMO time (hours)	2.8 (1.5-3.5)	1.3 (0-2.8)	1.7 (0-2.9)	26	<0.001
Post-operative outcomes					
Acute kidney injury	63 (54.8%)	33 (45.2%)	91 (43.1%)	4.1	0.12
Dialysis	23 (20.0%)	11 (15.1%)	26 (12.3%)	3.4	0.18
Stroke	5 (4.3%)	4 (5.5%)	3 (1.4%)	4.3	0.09
Bowel ischemia	2 (1.7%)	1 (1.4%)	3 (1.4%)	0.06	0.97
Digital ischemia	3 (2.6%)	1 (1.4%)	2 (0.9%)	1.4	0.5
PGD (any grade)	76 (66.1%)	35 (47.9%)	111 (52.6%)	7.6	0.02
PGD (grade 3)	22 (19.1%)	6 (8.2%)	22 (10.4%)	6.6	0.047
CLAD	14 (12.2%)	9 (12.3%)	32 (15.2%)	0.72	0.7
Post-transplant ECMO support	31 (27.0%)	5 (6.8%)	14 (6.6%)	22	<0.001
ICU stay (days)	10 (5-20)	7 (5-13)	7 (4-14)	6.2	0.046
Post-transplant ventilator (days)	2 (1-9)	2 (1-3)	2 (1-3)	10.5	0.005
Hospital stay (days)	21 (13-38)	17 (11-31)	16 (11-27)	9.9	0.007

Additionally, patients in the high-ME cohort experienced significantly more post-transplant complications, including elevated mortality risk and delayed recovery. High-ME patients showed a greater prevalence of PGD of any grade (66.1% vs. 47.9% (low-ME) vs. 52.6% (no ME), p = 0.02) and PGD Grade 3 (19.1% vs. 8.2% (low-ME) vs. 10.4% (no ME), p = 0.047). Rates of other early post-operative complications, such as AKI, dialysis requirement, stroke, bowel ischemia, and digital ischemia, did not differ significantly across ME groups. Post-transplant ECMO support was required more frequently in the high-ME cohort (27.0% vs. 6.8% (low-ME) vs. 6.6% (no ME), p < 0.001). These patients also had significantly longer intensive care unit (ICU) stays (median: 10 (5-20, IQR) vs. 7 (5-13, IQR, low-ME) vs. 7 (4-14, IQR, no ME) days, p = 0.046), post-transplant ventilator use (2 (1-9, IQR) vs. 2 (1-3, IQR, low-ME) vs. 2 (1-3, IQR, no ME) days, p = 0.005), and hospital stays (21 (13-38, IQR) vs. 17 (11-31, IQR, low-ME) vs. 16 (11-27, IQR, no ME) days, p = 0.007).

Survival analysis and impact of high ME use on survival

Predictors of survival were determined with univariate and multivariate Cox proportional hazards analysis (Table 3). An ME dosage of ≧ 0.1 mg was not significantly associated with overall survival (HR 1.13 (0.73-1.76, 95% CI), p = 0.59). Similarly, when opioid use was assessed as a continuous variable, higher dosages were not significantly associated with survival outcomes (HR 1.07 (0.71-1.60, 95% CI), p = 0.75). Other pre-transplant factors, including concurrent benzodiazepine use (HR 0.89 (0.58-1.38, 95% CI), p = 0.61), pre-transplant ventilator use (HR 0.66 (0.24-1.80, 95% CI), p = 0.42), pre-transplant HFNC use (HR 1.06 (0.56-1.98, 95% CI), p = 0.87), and pre-transplant ECMO use (HR 1.02 (0.54-1.93, 95% CI), p = 0.94), showed no significant associations with survival.

**Table 3:** Univariate and multivariate cox proportional hazards analysis to predict survival of recipients Hazard ratios (HR) for mortality were obtained using univariate and multivariate Cox proportional hazards analyses. Variables with p-values less than 0.05 in the univariate Cox proportional hazards analysis were included in the multivariate analysis. Etiology of lung failure (other): sarcoidosis, hypersensitivity pneumonitis, cystic fibrosis, bronchiectasis, obliterative bronchiolitis, bronchoalveolar carcinoma, primary ciliary dyskinesia ALT, Alanine aminotransferase; AST, aspartate aminotransferase; BUN, blood urea nitrogen; CI, confidence interval; COPD, chronic obstructive pulmonary disease; COVID-19, coronavirus disease 2019; ECMO, extracorporeal membrane oxygenation; FFP, fresh frozen plasma; HFNC, high-flow nasal cannula; HR, Hazard ratio; INR, international normalized ratio; ME, morphine equivalent; PAH, pulmonary artery hypertension; Plt, platelets; PGD, primary graft dysfunction; PRA, panel reactive antibody; pRBCs, packed red blood cells; VA-ECMO, venoarterial-extracorporeal membrane oxygenation; WBC, white blood cell

Variable	Univariate analysis			Multivariate analysis		
	HR	95% CI	p-value	HR	95% CI	p-value
Recipient factors						
Female	1.07	0.71-1.61	0.74	-		
Body mass index	1.04	0.99-1.09	0.12	-		
Body surface area	1.10	0.46-2.60	0.83	-		
Smoking history	1.05	0.70-1.57	0.81	-		
Hypertension	1.07	0.71-1.60	0.74	-		
Diabetes	1.40	0.93-2.12	0.11	-		
Concurrent benzodiazepine use	0.89	0.58-1.38	0.61	-		
Pre-transplant ventilator use	0.66	0.24-1.80	0.42	-		
Pre-transplant HFNC use	1.06	0.56-1.98	0.87	-		
Pre-transplant ECMO use	1.02	0.54-1.93	0.94	-		
Bilateral lung transplant	0.58	0.39-0.87	0.008	0.54	0.36-0.82	0.004
ME use within 30 days (continuous variable)	1.07	0.71-1.60	0.75	-		
ME >0.1 mg within 30 days	1.13	0.73-1.76	0.59	-		
Etiology of lung failure						
Interstitial lung disease	1.30	0.86-1.96	0.21	-		
COVID-19	0.76	0.40-1.43	0.39	-		
COPD	1.26	0.79-2.02	0.33	-		
PAH	0.54	0.17-1.70	0.29	-		
Other	0.79	0.47-1.28	0.32	-		
Laboratory						
Hemoglobin	0.99	0.91-1.07	0.78	-		
WBC	0.98	0.93-1.03	0.36	-		
Platelets	1.00	0.99-1.00	0.96	-		
Sodium	1.03	0.97-1.09	0.37	-		
BUN	1.01	0.98-1.03	0.63	-		
Creatinine	2.70	1.22-6.00	0.01	2.66	1.16-6.14	0.02
AST	1.01	1.00-1.02	0.048	1.01	0.99-1.02	0.08
ALT	1.01	0.99-1.02	0.16	-		
Albumin	0.68	0.47-0.98	0.04	0.64	0.43-0.95	0.03
Total bilirubin	1.19	0.91-1.57	0.22	-		
INR	1.25	0.45-3.49	0.67	-		
PRA	1.12	0.74-1.68	0.59	-		
Donor						
Age	1.01	0.99-1.03	0.10	-		
Female	1.16	0.76-1.77	0.48	-		
Cause of death						
Anoxia	0.99	0.65-1.50	0.96	-		
Head trauma	0.91	0.60-1.39	0.66	-		
Other	1.14	0.72-1.82	0.57	-		
Intra-operative outcomes						
Operative time	0.97	0.87-1.07	0.52	-		
Intra-op blood transfusion						
pRBCs	0.99	0.95-1.05	0.88	-		
FFP	1.01	0.93-1.09	0.89	-		
Plt	1.03	0.90-1.19	0.65	-		
Ischemic time	0.93	0.82-1.05	0.24	-		
VA-ECMO use	1.10	0.73-1.67	0.65	-		
VA-ECMO time	1.03	0.93-1.15	0.55	-		

**Figure 1:**
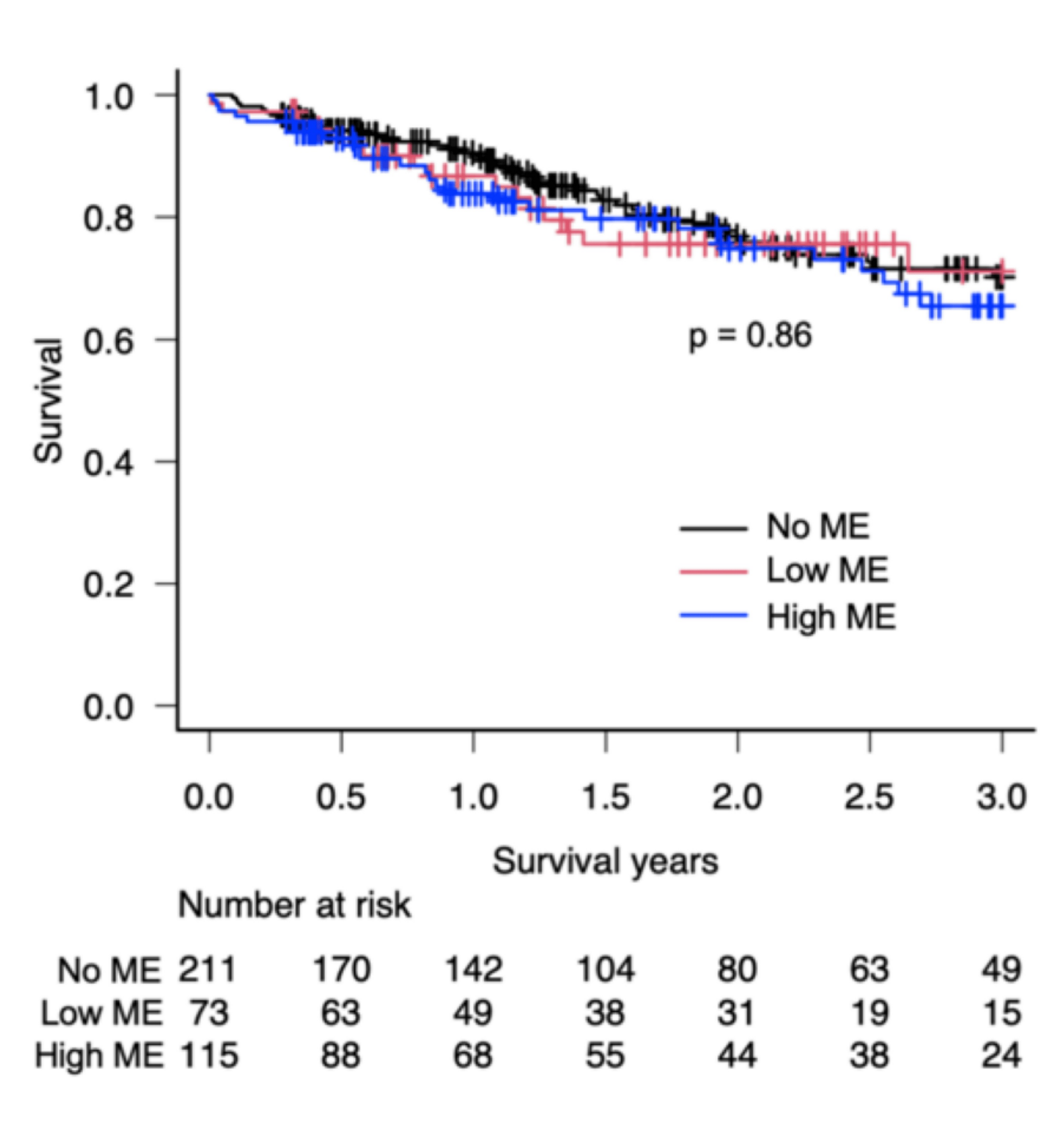
Overall survival of high-ME, low-ME, and no-ME patients after lung transplantation Kaplan-Meier survival curves compare overall survival between patients who received ME ≧ 0.1 mg within 30 days prior to lung transplantation (high-ME), ME < 0.1 mg (low-ME), and no ME (no-ME). The blue line represents the high-ME group, the red line represents the low-ME group, and the black line represents the no-ME group. The p-value of 0.86 indicates no significant difference in overall survival among the three groups. The numbers at risk at different time points are displayed below the graph. One-year survival is 83.8% (high-ME), 86.7% (low-ME), and 90.5% (no-ME). ME, morphine equivalent

Instead, multivariate analysis identified bilateral lung transplantation (HR 0.54 (0.36-0.82, 95% CI), p = 0.004) and higher serum albumin levels (HR 0.64 (0.43-0.95, 95% CI), p = 0.03) as independent predictors of improved prognosis, while elevated serum creatinine was associated with worse outcomes (HR 2.66 (1.16-6.14, 95% CI), p = 0.02). Kaplan-Meier survival curves show survival by preoperative opioid group: high-ME, low-ME, and no ME (Figure 1). There was no significant difference in one-year survival (83.8% vs. 86.7% (low-ME) vs. 90.5% (no ME), p = 0.86) and overall survival (p = 0.86) among the three groups (Figure 1).

Predictors of early postoperative complications

Univariate logistic regression analysis found that the risk of developing PGD of any grade (OR 1.84 (1.17-2.89, 95% CI), p = 0.008) (Table 4) and PGD Grade 3 (OR 2.16 (1.18-3.97, 95% CI), p = 0.01) (Table 5) was significantly higher in the high-ME cohort. However, in the multivariate logistic regression analysis, there was no significant association between high-ME dose and PGD of any grade (OR 1.58 (0.96-2.59, 95% CI), p = 0.07) or PGD Grade 3 (OR 0.71 (0.30-1.68, 95% CI), p = 0.43). Pulmonary artery hypertension as the etiology of lung failure (HR 7.70 (1.73-34.4, 95% CI), p = 0.007) and longer median ischemic times (HR 1.19 (1.08-1.32, 95% CI), p < 0.001) were independent risk factors for PGD of any grade in the multivariate logistic regression analysis. Lower serum albumin was an independent risk factor for developing PGD Grade 3 in the multivariate analysis (OR 0.51 (0.28-0.91, 95% CI), p = 0.02). Although pre-transplant ventilator use was an independent risk factor for PGD of any grade (OR 2.50 (1.12-5.56, 95% CI), p = 0.03) (Table 4), it was not associated with PGD Grade 3 (Table 5).

**Table 4:** Univariate and multivariate logistic regression analysis to predict PGD in any grade Odds ratios (OR) for primary graft dysfunction were obtained using univariate and multivariate logistic regression analyses. Variables with p-values less than 0.05 in the univariate logistic regression analysis were included in the multivariate analysis. Etiology of lung failure (other): sarcoidosis, hypersensitivity pneumonitis, cystic fibrosis, bronchiectasis, obliterative bronchiolitis, bronchoalveolar carcinoma, primary ciliary dyskinesia ALT, alanine aminotransferase; AST, aspartate aminotransferase; BUN, blood urea nitrogen; CI, confidence interval; COPD, chronic obstructive pulmonary disease; COVID-19, coronavirus disease 2019; ECMO, extracorporeal membrane oxygenation; FFP, fresh frozen plasma; HFNC, high-flow nasal cannula; INR, international normalized ratio; ME, morphine equivalent; OR, Odds ratio; PAH, pulmonary artery hypertension; Plt, platelets; PGD, primary graft dysfunction; PRA, panel reactive antibody; pRBCs, packed red blood cells; VA-ECMO, venoarterial-extracorporeal membrane oxygenation; WBC, white blood cell

Variable	Univariate analysis			Multivariate analysis		
	OR	95% CI	p-value	OR	95% CI	p-value
Recipient factors						
Female	1.15	0.77-1.72	0.49	-		
Body mass index	1.00	0.96-1.05	0.89	-		
Body surface area	0.92	0.41-2.05	0.83	-		
Smoking history	1.08	0.73-1.60	0.71	-		
Hypertension	1.20	0.81-1.79	0.36	-		
Diabetes	0.79	0.52-1.22	0.29	-		
Concurrent benzodiazepine use	1.32	0.86-2.03	0.20	-		
Pre-transplant ventilator use	3.26	1.52-7.01	0.002	2.50	1.12-5.56	0.03
Pre-transplant HFNC use	2.00	1.12-3.57	0.02	-		
Pre-transplant ECMO use	1.75	0.87-3.49	0.12	-		
Bilateral lung transplant	1.75	1.17-2.64	0.007	0.89	0.52-1.52	0.66
ME use within 30 days (continuous variable)	1.31	0.88-1.95	0.19	-		
ME ≧ 0.1 mg within 30 days	1.84	1.17-2.89	0.008	1.58	0.96-2.59	0.07
Etiology of lung failure						
Interstitial lung disease	0.78	0.52-1.17	0.23	-		
COVID-19	1.53	0.82-2.86	0.19	-		
COPD	0.58	0.35-0.96	0.03	-		
PAH	7.72	1.77-33.7	0.007	8.42	1.88-37.6	0.005
Other	1.16	0.74-1.82	0.51	-		
Laboratory						
Hemoglobin (g/dL)	0.97	0.89-1.05	0.42	-		
WBC (1,000/mm^3^)	0.96	0.91-1.01	0.16	-		
Platelets (1,000/mm^3^)	1.00	0.99-1.00	0.59	-		
Sodium (mEq/L)	1.02	0.96-1.08	0.62	-		
BUN (mg/dL)	1.01	0.99-1.04	0.36	-		
Creatinine (mg/dL)	1.64	0.75-3.63	0.22	-		
AST (U/L)	0.99	0.98-1.01	0.36	-		
ALT (U/L)	1.00	0.99-1.01	0.99	-		
Albumin (g/dL)	0.85	0.58-1.24	0.39	-		
Total bilirubin (mg/dL)	1.01	0.71-1.44	0.96	-		
INR	0.78	0.27-2.19	0.63	-		
PRA	1.13	0.75-1.69	0.57	-		
Donor						
Age (years)	1.02	1.00-1.03	0.04	-		
Female	0.77	0.50-1.17	0.22	-		
Cause of death						
Anoxia	1.10	0.73-1.64	0.65	-		
Head trauma	0.76	0.50-1.15	0.19	-		
Other	1.25	0.79-1.99	0.34	-		
Intra-operative outcomes						
Operative time (hours)	1.01	0.92-1.12	0.76	-		
Intra-op blood transfusion						
pRBCs (units)	1.07	1.01-1.13	0.02	-		
FFP (units)	1.11	1.00-1.22	0.04	-		
Plt (units)	1.13	0.96-1.34	0.15	-		
Ischemic time (hours)	1.23	1.10-1.36	<0.001	1.18	1.06-1.31	0.002
VA-ECMO use	2.04	1.35-3.08	<0.001	1.42	0.84-2.40	0.19
VA-ECMO time (hours)	1.10	0.99-1.23	0.08	-		

**Table 5:** Univariate and multivariate logistic regression analysis to predict PGD Grade 3 Odds ratios (OR) for primary graft dysfunction were obtained using univariate and multivariate logistic regression analyses. Variables with p-values less than 0.05 in the univariate logistic regression analysis were included in the multivariate analysis. Etiology of lung failure (other): sarcoidosis, hypersensitivity pneumonitis, cystic fibrosis, bronchiectasis, obliterative bronchiolitis, bronchoalveolar carcinoma, primary ciliary dyskinesia ALT, Alanine aminotransferase; AST, aspartate aminotransferase; BUN, blood urea nitrogen; CI, confidence interval; COPD, chronic obstructive pulmonary disease; COVID-19, coronavirus disease 2019; ECMO, extracorporeal membrane oxygenation; FFP, fresh frozen plasma; HFNC, high-flow nasal cannula; INR, international normalized ratio; ME, morphine equivalent; OR, Odds ratio; PAH, pulmonary artery hypertension; Plt, platelets; PGD, primary graft dysfunction; PRA, panel reactive antibody; pRBCs, packed red blood cells; VA-ECMO, venoarterial-extracorporeal membrane oxygenation; WBC, white blood cell

Variable	Univariate analysis			Multivariate analysis		
	OR	95% CI	p-value	OR	95% CI	p-value
Recipient factors						
Female	1.28	0.71-2.33	0.41	-		
Body mass index (kg/m^2^)	1.01	0.94-1.07	0.85	-		
Body surface area (m^2^)	1.20	0.36-3.98	0.77	-		
Smoking history	0.93	0.51-1.68	0.81	-		
Hypertension	1.02	0.56-1.85	0.96	-		
Diabetes	0.97	0.51-1.85	0.93	-		
Concurrent benzodiazepine use	1.33	0.72-2.46	0.36	-		
Pre-transplant ventilator use	1.19	0.47-2.98	0.72	-		
Pre-transplant HFNC use	0.71	0.29-1.75	0.46	-		
Pre-transplant ECMO use	6.37	3.09-13.2	<0.001	3.50	0.83-14.7	0.09
Bilateral lung transplant	1.34	0.71-2.52	0.36	-		
ME use within 30 days (continuous variable)	1.50	0.83-2.73	0.18	-		
ME ≧ 0.1 mg within 30 days	2.16	1.18-3.97	0.01	0.71	0.30-1.68	0.43
Etiology of lung failure						
Interstitial lung disease	0.87	0.47-1.62	0.65	-		
COVID-19	2.75	1.32-5.73	0.007	0.70	0.22-2.27	0.55
COPD	0.34	0.12-0.96	0.05	-		
PAH	1.25	0.35-4.42	0.73	-		
Other	1.05	0.55-2.04	0.81	-		
Laboratory						
Hemoglobin (g/dL)	0.88	0.78-1.00	0.05	-		
WBC (1,000/mm^3^)	1	0.93-1.08	0.98	-		
Platelets (1,000/mm^3^)	1	0.99-1.00	0.07	-		
Sodium (mEq/L)	1.05	0.97-1.15	0.25	-		
BUN (mg/dL)	1.02	0.99-1.06	0.17	-		
Creatinine (mg/dL)	2.51	0.84-7.48	0.10	-		
AST (U/L)	1.01	1.00-1.03	0.03	-		
ALT (U/L)	1.00	0.99-1.02	0.53	-		
Albumin (g/dL)	0.38	0.22-0.65	<0.001	0.51	0.28-0.91	0.02
Total bilirubin (mg/dL)	1.76	1.15-2.68	0.009	1.35	0.83-2.18	0.22
INR	2.73	0.80-9.32	0.11	-		
PRA	1.54	0.85-2.79	0.16	-		
Donor						
Age (years)	1.01	0.99-1.04	0.28	-		
Female	1.83	0.99-3.34	0.051	-		
Cause of death						
Anoxia	1.24	0.68-2.25	0.47	-		
Head trauma	0.80	0.42-1.52	0.49	-		
Other	0.98	0.49-1.96	0.96	-		
Intra-operative outcomes						
Operative time (hours)	1.25	1.08-1.44	0.003	1.02	0.83-1.25	0.85
Intra-op blood transfusion						
pRBCs (units)	1.14	1.07-1.20	<0.001	1.03	0.86-1.23	0.79
FFP (units)	1.19	1.09-1.31	<0.001	1.05	0.85-1.30	0.67
Plt (units)	1.42	1.19-1.71	<0.001	1.06	0.75-1.50	0.73
Ischemic time (hours)	0.99	0.90-1.09	0.81	-		
VA-ECMO use	1.64	0.85-3.16	0.14	-		
VA-ECMO time (hours)	1.05	0.91-1.20	0.50	-		

## Discussion

Opioids are frequently prescribed to patients with end-stage lung disease awaiting lung transplantation to manage chronic pain and dyspnea. This study investigated how preoperative opioid use in lung transplant recipients influences early post-transplant recovery and mortality risk. In our patient cohort, 188 patients (47.1%) had an opioid prescription within 30 days before lung transplantation; 115 (61.2%) were categorized as high-ME (ME ≧ 0.1 mg), and 73 (38.8%) as low-ME (ME < 0.1 mg). Although one-year and overall survival were comparable across groups, high-ME patients had higher rates of PGD in any grade, and PGD Grade 3 specifically. High-ME patients also had prolonged ventilator use, ICU stay, and hospital length of stay. The high-ME cohort had higher lung allocation scores, more pre-transplant ECMO support, and greater rates of bilateral lung transplantation, indicating they were likely more critically ill at baseline, which may influence the differences observed between groups. These findings suggest that preoperative opioid use above a 0.1 mg ME threshold warrants close assessment to reduce the risk of both short- and long-term complications.

Previous studies indicate that preoperative opioid use at high doses is associated with increased mortality risk in patients with severe lung disease or lung transplantation candidates [20,21]. Conversely, some research has reported no significant differences in retransplant-free survival or chronic lung allograft dysfunction (CLAD) incidence among lung transplant recipients with preoperative opioid use [22]. Our analysis revealed no significant difference in one-year survival (83.8% (high-ME) vs. 86.7% (low-ME) vs. 90.5% (no ME), p = 0.86) or overall survival (p = 0.86) among the ME groups. These discrepancies may reflect study design differences. Prior research associating opioids with higher mortality examined use over longer preoperative periods, with a higher dosage threshold of 7 mg - potentially capturing cumulative survival effects of high opioid dosage that are not evident in our 30-day analysis [20,21].

Although survival outcomes did not differ significantly across ME groups, univariate logistic regression revealed that the high-ME cohort was at greater risk for PGD at any grade and PGD Grade 3 specifically. While the association was attenuated in the multivariate analysis, this likely reflects limitations in sample size. Notably, other potential confounding factors - including concurrent benzodiazepine use, pre-transplant ventilator use, and pre-transplant HFNC use - did not significantly predict survival or PGD Grade 3, suggesting that our findings are less likely to be explained by differences in pre-transplant illness severity. These findings indicate that opioid use ≧ 0.1 mg ME may contribute to PGD Grade 3 development. Given the established association between PGD and greater long-term mortality [23], this highlights the need for further research on the effects of opioid use. Although short-term survival rates were similar, our data suggest that transplant teams should carefully weigh the risks of perioperative complications and long-term outcomes when managing opioid prescriptions in lung transplant patients.

Another important finding from this study was that patients in the high-ME group experienced significantly longer durations of post-transplant ventilation, ICU stay, and hospitalization. These findings align with prior studies linking opioid sedation to extended ventilator use and hospital stay, including one study noting prolonged stays with a minimum dose of <1 mg/day, and another that reported similar findings in patients with a median dose of 31 mg [20,22]. While these studies evaluated higher opioid doses than our 0.1 mg ME cutoff, our findings indicate that even this lower dose may be sufficient to extend recovery times. Opioids, especially when used with medications like benzodiazepines, can suppress respiratory drive, delaying weaning from mechanical ventilation and increasing the risk of in-hospital rejection [20,24,25]. Additionally, opioid-induced sedation may increase the risk of delirium in mechanically ventilated patients, which may prolong ICU and hospital duration [26]. Extended postoperative recovery is linked to higher rates of ventilator-associated pneumonia, in-hospital mortality, and increased healthcare costs in lung transplant recipients [27-29]. While differences in outcomes between ME groups may be partially confounded by more severe baseline illness in the high-ME cohort, the association with longer recovery times remains notable. To reduce mortality risks and support rehabilitation, careful tapering of opioids and sedatives before transplantation may be beneficial. 

Preoperative opioid use presents additional challenges beyond the risks of increased mortality and delayed recovery. Patients may develop persistent opioid misuse or experience opioid-induced hyperalgesia following surgery, contributing to difficulties with adequate pain control. In our study, the high-ME group had a significantly higher maximum opioid dose at 90 days post-transplant, suggesting more persistent opioid use in the early post-transplant period. However, this difference was no longer significant by one year, indicating potential resolution over time. Despite these risks, opioids continue to be prescribed primarily to manage preoperative pain and facilitate mechanical ventilation. Effective perioperative pain management remains crucial, especially because postoperative pain itself is a predictor of postoperative delirium [9]. However, to minimize the risk of post-transplant opioid misuse, gradual weaning of opioids prior to lung transplantation may be beneficial [30]. Alternatively, transplant teams could explore multimodal approaches for preoperative pain management and hemodynamic stabilization, incorporating non-opioid analgesics [13]. Further research is needed to determine appropriate opioid use and dosing strategies to reduce complications such as postoperative delirium, while maintaining effective pain control. 

This study has several limitations. The single-center, retrospective design and methods used for patient selection may reduce the statistical power of our analysis and limit the generalizability of our findings. The high-ME cohort had higher rates of pre-transplant ECMO utilization and bilateral lung transplantation, indicating that those patients may have had more advanced pre-transplant illness, potentially influencing the outcomes observed. However, ECMO was not a significant predictor of survival or PGD Grade 3, and bilateral transplantation was associated with improved survival, not PGD Grade 3. Due to limited sample size, propensity score matching was not performed. While we included patients with an opioid prescription within 30 days prior to lung transplantation, prescription records may not accurately represent patients’ actual opioid consumption. Furthermore, data were not collected on preoperative psychosocial assessments, which may help identify individuals with underlying substance use disorders. These conditions may contribute to increased and more frequent opioid use, potentially affecting our findings. Lastly, although high-ME patients demonstrated similar one-year survival outcomes compared to other groups, the potential long-term consequences of preoperative opioid use beyond the first year after transplantation remain uncertain. These limitations may have reduced our ability to detect significant associations and limit the generalizability of our results. Unmeasured factors, such as psychosocial status and pain severity assessments, could also have influenced outcomes. Prospective studies with standardized evaluation of these factors are needed to clarify their impact. Despite these constraints, our study offers valuable insight into the possible effects of preoperative opioid use on lung transplant outcomes and identifies important areas for future research.

## Conclusions

In conclusion, our findings suggest a trend toward higher rates of PGD of any grade, PGD Grade 3, and prolonged postoperative recovery, including extended ventilator support and longer ICU and hospital stays, among lung transplant recipients receiving preoperative opioid doses ≥ 0.1 mg ME. While one-year survival did not differ significantly across groups, these results suggest that preoperative opioid use may still contribute to early post-transplant complications. Careful opioid weaning and alternative approaches to managing chronic pain and dyspnea should be considered by transplant teams to minimize perioperative risk, with the goal of optimizing pain control while avoiding unnecessary opioid exposure - rather than excluding such patients from transplant candidacy.
